# Prior viral infection primes cross-reactive CD8+ T cells that respond to mouse heart allografts

**DOI:** 10.3389/fimmu.2023.1287546

**Published:** 2023-12-08

**Authors:** M. Eyad Khorki, Tiffany Shi, Eileen E. Cianciolo, Ashley R. Burg, P. Chukwunalu Chukwuma, Jennifer L. Picarsic, Mary K. Morrice, E. Steve Woodle, Jonathan S. Maltzman, Autumn Ferguson, Jonathan D. Katz, Brian M. Baker, David A. Hildeman

**Affiliations:** ^1^ Division of Nephrology & Hypertension, Cincinnati Children’s Hospital Medical Center, Cincinnati, OH, United States; ^2^ Division of Immunobiology, Cincinnati Children’s Hospital Medical Center, Cincinnati, OH, United States; ^3^ Immunology Graduate Program, University of Cincinnati College of Medicine, Cincinnati, OH, United States; ^4^ Medical Scientist Training Program, University of Cincinnati College of Medicine, Cincinnati, OH, United States; ^5^ Department of Chemistry & Biochemistry and the Harper Cancer Research Institute, University of Notre Dame, Notre Dame, IN, United States; ^6^ Division of Pathology, Cincinnati Children’s Hospital Medical Center, Cincinnati, OH, United States; ^7^ Department of Pathology, University of Cincinnati College of Medicine, Cincinnati, OH, United States; ^8^ Division of Transplantation, Department of Surgery, University of Cincinnati College of Medicine, Cincinnati, OH, United States; ^9^ Department of Medicine, Stanford University, Palo Alto, CA, United States; ^10^ Geriatric Research and Education Clinical Center, Veterans Affairs (VA) Palo Alto Health Care System, Palo Alto, CA, United States

**Keywords:** transplant, cross-reactivity, TCR (T cell receptor), LCMV (lymphocytic choriomeningitis virus), transplantation, CD8^+^ T cell response, heterologous immunity

## Abstract

**Introduction:**

Significant evidence suggests a connection between transplant rejection and the presence of high levels of pre-existing memory T cells. Viral infection can elicit viral-specific memory T cells that cross-react with allo-MHC capable of driving allograft rejection in mice. Despite these advances, and despite their critical role in transplant rejection, a systematic study of allo-reactive memory T cells, their specificities, and the role of cross-reactivity with viral antigens has not been performed.

**Methods:**

Here, we established a model to identify, isolate, and characterize cross-reactive T cells using Nur77 reporter mice (C57BL/6 background), which transiently express GFP exclusively upon TCR engagement. We infected Nur77 mice with lymphocytic choriomeningitis virus (LCMV-Armstrong) to generate a robust memory compartment, where quiescent LCMV-specific memory CD8^+^ T cells could be readily tracked with MHC tetramer staining. Then, we transplanted LCMV immune mice with allogeneic hearts and monitored expression of GFP within MHC-tetramer defined viral-specific T cells as an indicator of their ability to cross-react with alloantigens.

**Results:**

Strikingly, prior LCMV infection significantly increased the kinetics and magnitude of rejection as well as CD8^+^ T cell recruitment into allogeneic, but not syngeneic, transplanted hearts, relative to non-infected controls. Interestingly, as early as day 1 after allogeneic heart transplant an average of ~8% of MHC-tetramer^+^ CD8^+^ T cells expressed GFP, in contrast to syngeneic heart transplants, where the frequency of viral-specific CD8^+^ T cells that were GFP^+^ was <1%. These data show that a significant percentage of viral-specific memory CD8^+^ T cells expressed T cell receptors that also recognized alloantigens *in vivo*. Notably, the frequency of cross-reactive CD8^+^ T cells differed depending upon the viral epitope. Further, TCR sequences derived from cross-reactive T cells harbored distinctive motifs that may provide insight into cross-reactivity and allo-specificity.

**Discussion:**

In sum, we have established a mouse model to track viral-specific, allo-specific, and cross-reactive T cells; revealing that prior infection elicits substantial numbers of viral-specific T cells that cross-react to alloantigen, respond very early after transplant, and may promote rapid rejection.

## Introduction

T cell receptors (TCRs) recognize peptides in the context of major histocompatibility complex (MHC) molecules. During thymic development, processes of positive and negative selection generate T cells, each bearing individual TCRs that are restricted to self-MHC and that, collectively, have the capability of recognizing a broad array of non-self peptides (1). Despite their self-MHC restriction and fine specificity for foreign peptide epitopes, T cell recognition of non-self MHC molecules, so-called “allo”-recognition, is extremely robust (2, 3). The long-established paradigm is that TCRs on alloreactive T cells are either allo-MHC centric and thus bind largely independent of peptide (the “high-determinant density model”) or allo-peptide centric and thus bind largely independent of MHC (the “multiple binary complex model”) (4, 5). The models differ in the presumed way the TCR interacts with allo-MHC and allo-peptide, but both imply low specificity and extensive cross-reactivity. However, our data, as well as that of others, has suggested that some alloreactive T cells recognize features of both allo-MHC and allo-peptide, a phenomenon referred to as allo-specificity (6–11). Thus, current data suggest that alloreactive T cells can display similar levels of specificity towards their targets as do conventional T cells.

Understanding allo-specificity has been hampered by the lack of a systematic approaches to identify T cells bearing allo-specific TCRs and as well as the peptide/MHC molecules they recognize. Such information is important, because CD8^+^ T cells are the primary drivers of acute cellular rejection (ACR) (12). Although recipient alloreactive T cells can recognize donor-derived epitopes on self-HLA, via a process called indirect recognition, in general, it is thought that ACR is driven by direct recognition of donor HLA on transplanted donor tissues (12). Intriguing recent data has also suggested a connection between alloreactivity and prior (or existing) viral infection. Notably, some studies have shown the presence of viral-specific T cells present within allografts (13, 14). Others have shown that high levels of endogenous memory T cells prior to transplant are associated with graft rejection (15–17). Two non-mutually exclusive concepts have been put forward to explain this phenomenon. One concept is that viral-specific T cells are activated non-specifically during rejection, referred to as “bystander” activation (18). Another concept is that, depending upon the viral infection, T cells with viral-specificity also possess alloreactivity, referred to as “cross-reactivity” or “heterologous immunity” (18).

Several studies in mice and humans have provided strong evidence supporting the second concept. Early pioneering work in mice showed that prior viral infections can disrupt tolerance to skin allografts (19) and elicit robust populations of viral-specific T cells with allospecificity (19–22). Importantly, purification and adoptive transfer of viral-specific CD8^+^ T cells was capable of driving allograft rejection in mice (20). While prior infection of BL/6 mice with lymphocytic choriomeningitis virus (LCMV) was capable of eliciting viral-specific CD8^+^ T cells with allo-specific cross-reactivity (19, 20) to Balb/c alloantigens. However, such phenomena was not observed in BL/6 mice with prior mouse polyoma virus (mPyV) infection (23) in response to C3H alloantigens. Nonetheless, several studies in humans show that viral-specific T cells also cross-react with alloantigens (7, 24–26). One study showed that MHC-tetramer purified, viral specific CD8^+^ T cells derived from human PBMCs readily recognized allogeneic cells (24). Another group has shown a strong association between the pre-transplant presence of clonally expanded CMV-specific CD8^+^ T cells and subsequent allograft rejection, including overlapping TCRβ chain sequences in both CMV-specific as well as alloreactive cells in the rejecting allografts (13). Altogether, a consensus is emerging that high levels of memory T cells, including those with viral specificities, predispose for allograft rejection, and that many of these viral-specific T cells are in fact allo-specific. However, a systematic study of allograft resident T cells and their potential for cross-reactivity has not been performed. This is of particular importance as we start to see widespread use of viral-specific T cells (VSTs) therapy to treat various viral infections in solid organ transplantation.

Here, we developed a model system to examine the impact of prior viral infection on alloreactivity. We took advantage of Nur77-GFP transgenic mice, whose GFP expression is restricted to T and B cells receiving signals through their TCRs and BCRs, respectively (27). Importantly, the level of GFP within responding cells is directly correlated with TCR strength of signal (27). We found that prior viral infection dramatically increases the recruitment of CD8^+^ T cells to allogeneic heart allografts. We found a large proportion of viral-specific CD8^+^ T cells rapidly expressed GFP within allogeneic, but not syngeneic, heart allografts early after transplantation. Interestingly, the level of cross-reactivity varied between viral epitopes, suggesting the potential for cross-reactivity is shaped by the pre-existing naïve repertoire. Together, we think this model will allow for systematic analysis and understanding of not only viral-specific T cells and their potential for cross-reactivity, but also identification of TCRs from allo-specific T cells and facilitation of approaches to identify the peptide/MHC complexes they recognize and mechanism of recognition.

## Results

### LCMV-immune mice have earlier and more robust CD8^+^ T cell infiltration into heart allografts as compared to LCMV-naïve mice

**Figure 1:**
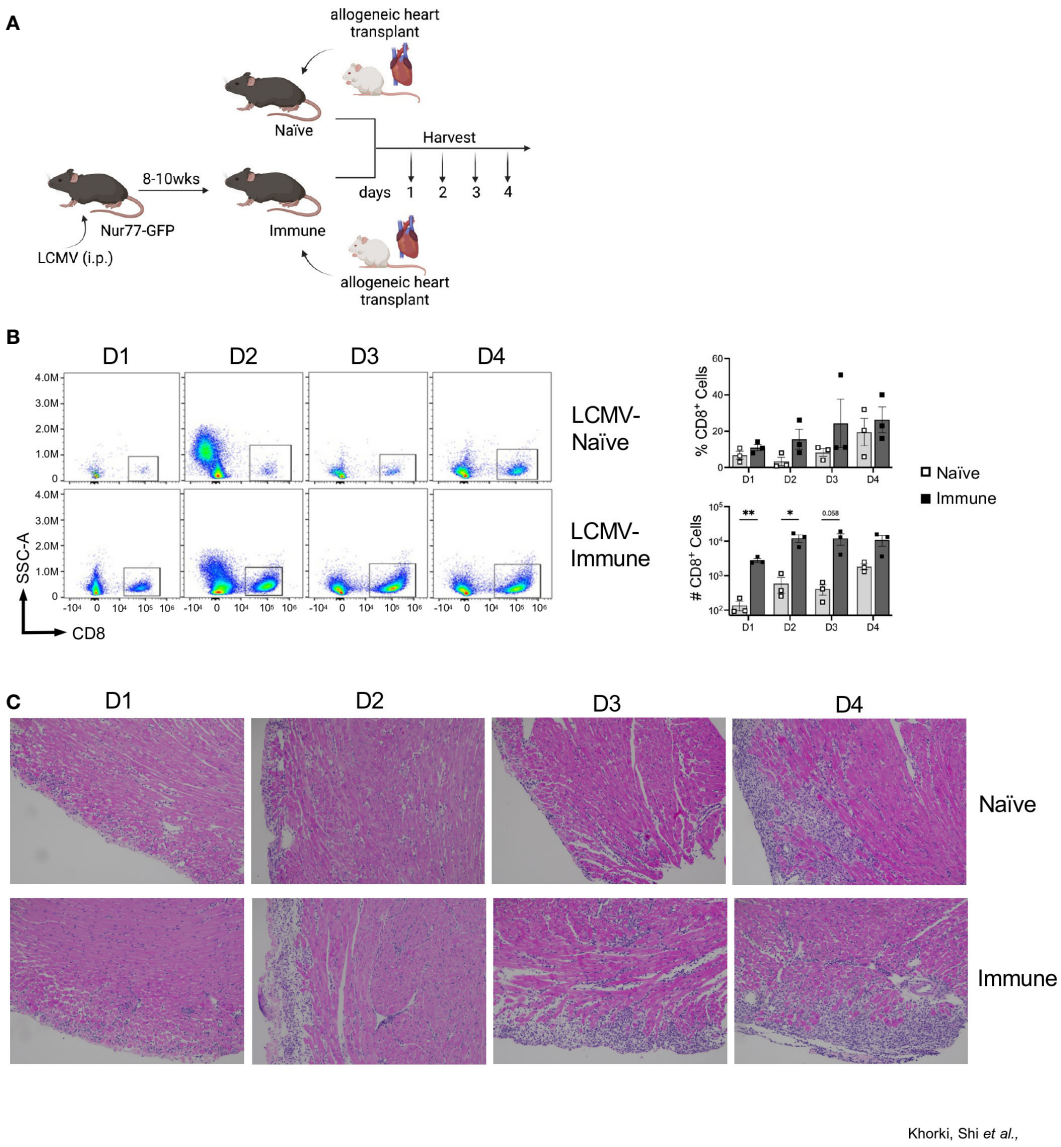
Prior viral infection increases recruitment of CD8^+^ T cells into heart allografts. **(A)** Experimental outline showing allogeneic heterotopic heart transplantation in either LCMV-naïve or LCMV-immune mice, followed by sample collection at days 1 through 4 after transplantation Images were produced in BioRender ^(TM)^. **(B)** CD8^+^ infiltration into heart allografts at days 1 through 4 after transplantation. **(C)** Representative H&E staining of heart allografts. Bar graphs show mean ± SEM. Statistical analyses were performed with student t-test between indicated groups (* p<0.05, ** p< 0.005).

LCMV-Armstrong infection of mice elicits a robust T cell response that drives clearance of the virus by 8-10 days after infection, leaving behind a large pool of quiescent memory CD8^+^ T cells that persist for the life of the animal (28). To determine the impact of a pre-existing, quiescent memory T cell population on the early allogenic response, we developed a murine heart transplant model in which we can track the direct activation of these memory T cells against the allograft. Heart allografts from Balb/c mice were heterotopically transplanted into Nur77-GFP transgenic C57BL/6 recipient mice that were either LCMV- naïve or LCMV-immune, which were given LCMV 8-10 weeks prior (**Figure 1A**). On days 1 through 4 after transplant, hearts were collected and CD8^+^ T cell infiltration was assessed by flow cytometry (**Figure 1A**). As early as day 1 after transplant, LCMV-immune mice had a remarkable infiltration of CD8^+^ T cells into the allograft (**Figure 1B**, **Supplemental Figure 1**). Overall, there were more infiltrating CD8^+^ T cells in immune compared to naïve mice on days 1-4 post-transplantation, with significant differences seen at days 1 and 2 after transplantation (**Figure 1B**). These data show that immune mice have increased kinetics and magnitude of CD8^+^ T cell infiltration into the allograft relative to naïve mice.

To confirm the flow cytometric data, we performed histological analyses via H&E staining on transplanted Balb/c hearts. Histologic staining showed progressively increased lymphocytic infiltration from day 1 through 4 after transplantation, which was substantially more pronounced in immune mice (**Figure 1C**). Blinded histologic analyses and scoring on H&E-stained tissue sections reflected the flow cytometric data as well, with earlier and progressively increased histologic rejection scores seen in the immune mice (**Table 1**). For example, rejection scores were more severe in immune mice at day 2 as compared to naïve mice at day 3. Further, histologic analyses revealed that immune mice had infiltrating neutrophils and eosinophils as early as day 2 after transplant, indicating worsened pathology as compared to naïve mice at day 4 post-transplant, which only had a few neutrophils and eosinophils present. Naïve mice had small foci of epicardial damage at days 1 and 2 after transplant which is expected due to the ischemia/reperfusion injury inherent in the heart transplant procedure. While the overall histology scores were the same by day 4 after transplant, the immune mice had more severe myocyte tissue damage and more inflammatory infiltration (**Table 1**). Overall, the presence of large numbers of memory T cells prior to transplantation accelerates both the recruitment of CD8^+^ T cell into heart allografts and allograft tissue damage.

**Table 1:** Histologic scoring for rejection on naïve and immune mice receiving allogeneic heterotopic cervical heart transplants using the 1990 and 2004 International Society of Heart and Lung Transplantation (ISHLT) criteria.

Status	PTD	Myocyte Tissue Damage		Inflammatory Infiltration					Rejection Score	
		Multifocal	Intensity	Interstitial	Epicardial	Endocardial	Eosinophilic	Neutrophilic	1990	2004
Naive	D1*	yes^	minimal	minimal	minimal	no	no	yes (epicardial)	1A-3A	1R-2R
	D2*	yes^	minimal	minimal	minimal	no	no	no	1A-3A	2R
	D3	yes	moderate	minimal	minimal	yes^#^	no	no	3A	2R
	D4	yes	severe	moderate	moderate	yes	yes (few, epicardial)	yes (few, epicardial)	3B	3R
Immune	D1	yes^	mild	mild	mild	yes	no	no	1B-3A	1R-2R
	D2	yes	moderate	moderate	severe	yes	yes	yes	3A-3B	2R-3R
	D3	yes	severe	severe	severe	yes	yes	yes	3B	3R
	D4	yes	severe	severe	severe	yes	yes	yes	3B	3R

*tissue damage and infiltration are minimal within minute foci and limited to epicardium; may be reflective of ischemic procedural effect ^small foci at epicardium #likely response to ischemia/reperfusion PTD, post-transplant day.

### Prior viral infection results in early recruitment of alloreactive CD44^hi^CD8^+^ T cells into heart allografts

**Figure 2:**
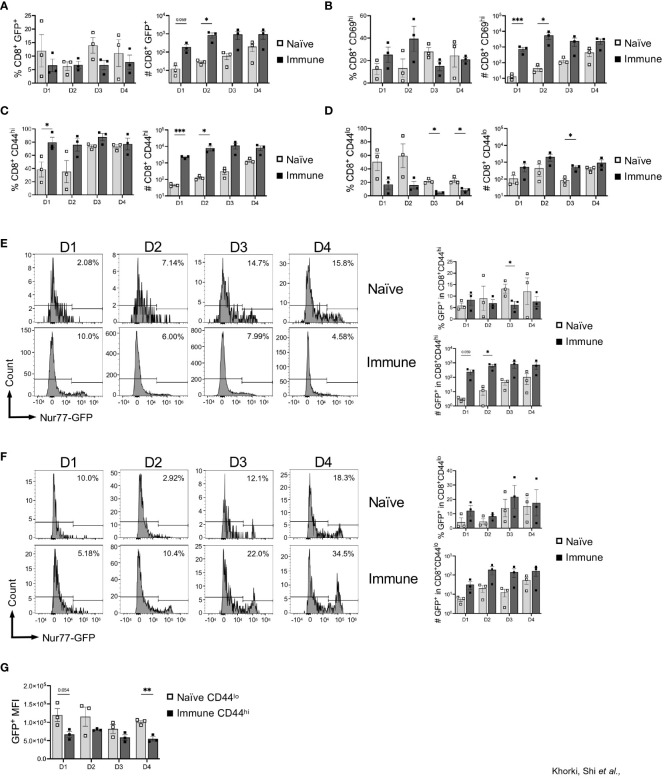
Prior viral infection promotes preferential recruitment of pre-existing memory CD8^+^ T cells into heart allografts. Graphs show the frequency and total numbers of graft-infiltrating CD8^+^ T cells that are **(A)** Nur77^+^, **(B)** CD69^+^, and **(C)** CD44^hi^, and **(D)** CD44^lo^. Dot plots show temporal expression of Nur77-GFP after gating on **(E)** CD8^+^CD44^hi^ and **(F)** CD8^+^CD44^lo^ T cells. **(G)** Mean fluorescence intensity (MFI) of GFP^+^ cells between naïve CD44^lo^ cells and immune CD44^hi^ cells. Bar graphs show mean ± SEM. Statistical analyses were performed with student t-test between indicated groups (* p<0.05, ** p< 0.005, *** p<0.0005).

Taking advantage of the Nur77-GFP transgenic mice, we next assessed GFP expression in the graft-infiltrating CD8^+^ T cells. Given that GFP expression is limited to those CD8^+^ T cells receiving very recent TCR signals, we considered GFP^+^ T cells in the allograft to be alloreactive. On average, approximately 10% of the CD8^+^ T cells in the graft were alloreactive (GFP^+^) from days 1 through 4 after transplant in both naïve and immune mice (**Figure 2A**). Although no significant difference was seen in the percentage of alloreactive CD8^+^ T cells recruited to the allograft between the naïve and immune mice, immune mice had dramatically increased numbers of alloreactive cells due to a more robust CD8^+^ T cell response (**Figure 2A**). Expression of CD69, measured as an independent marker of activation, was also found to be increased in the number, but not the frequency, of CD8^+^ T cells in immune mice (**Figure 2B**, **Supplemental Figure 2**). Given the transient nature of Nur77-GFP and CD69 expression, the extent of early activated CD8^+^ T cells detected is likely an underestimate.

To determine whether this increased infiltration was explained by an increased presence of memory T cells, we examined expression of CD44 on graft infiltrating CD8^+^ T cells. Although expression of CD44, in this instance could also reflect activation within the allograft, we found that significantly less CD8^+^ T cells in naïve mice were CD44^hi^ (40-80%), while the majority of the CD8^+^ T cells in immune mice were CD44^hi^ (80-90%) (**Figure 2C**). Consistent with their delayed response, the frequency and number of CD44^hi^ cells in naïve mice increased from day 1 to day 4 (**Figure 2C**). Additionally, we saw that, compared to days 1 and 2, at days 3 and 4 after transplantation, there was a large increase in the percentage of CD8^+^CD44^hi^ cells after transplantation in naïve mice, indicating the time it takes for cells to transition from CD44^lo^ to CD44^hi^ after antigen stimulation (**Figure 2C**). Further, recruitment of CD44^lo^ cells into allografts of naïve mice persisted for days, and the overall numbers of CD44^lo^ and CD44^hi^ cells were both increased in immune mice (**Figures 2C, D**). Overall, there was preferential early recruitment of memory CD8^+^ T cells into the allograft in mice with a prior LCMV infection.

Next, we determined whether allograft infiltrating CD8^+^ T cells are recognizing alloantigen in the graft, by assessing expression of GFP as an indicator of TCR stimulation in those CD8^+^ T cells in relation to CD44 expression. Interestingly, we found minimal differences in the percentage of CD8^+^CD44^hi^ that were GFP^+^ between naïve and immune mice, although the overall numbers of CD8^+^CD44^hi^GFP^+^ were significantly increased early after transplant in immune mice (**Figure 2E**). Further, immune mice also had an increase in CD8^+^CD44^lo^ cells that were GFP^+^ compared to naïve mice (**Figure 2F**). As the level of GFP per cell is proportional to TCR signal strength, we assessed the mean fluorescence intensity of GFP in CD8^+^ T cells in naïve vs immune mice (27, 29). We focused on comparing CD44^lo^ T cells in naïve mice (which should represent the naïve T cell response to alloantigen) to CD44^hi^ T cells in immune mice (which, at least in the first two days, should be largely comprised of cross-reactive T cells). In general, we found that the CD44^lo^ T cells in naïve mice had a higher GFP MFI compared to CD44^hi^ T cells from immune mice, suggesting that naïve T cells responding to alloantigen are receiving stronger TCR signals (**Figure 2G**). Thus, in addition to recruitment and activation of pre-existing memory CD8^+^ T cells into heart allografts, immune mice also had increased numbers of allo-activated naïve CD8^+^ T cells, although they may be of lower affinity.

### Increased recruitment of CD8^+^ T cells into heart allografts in mice with prior viral infection is an allogeneic response

**Figure 3:**
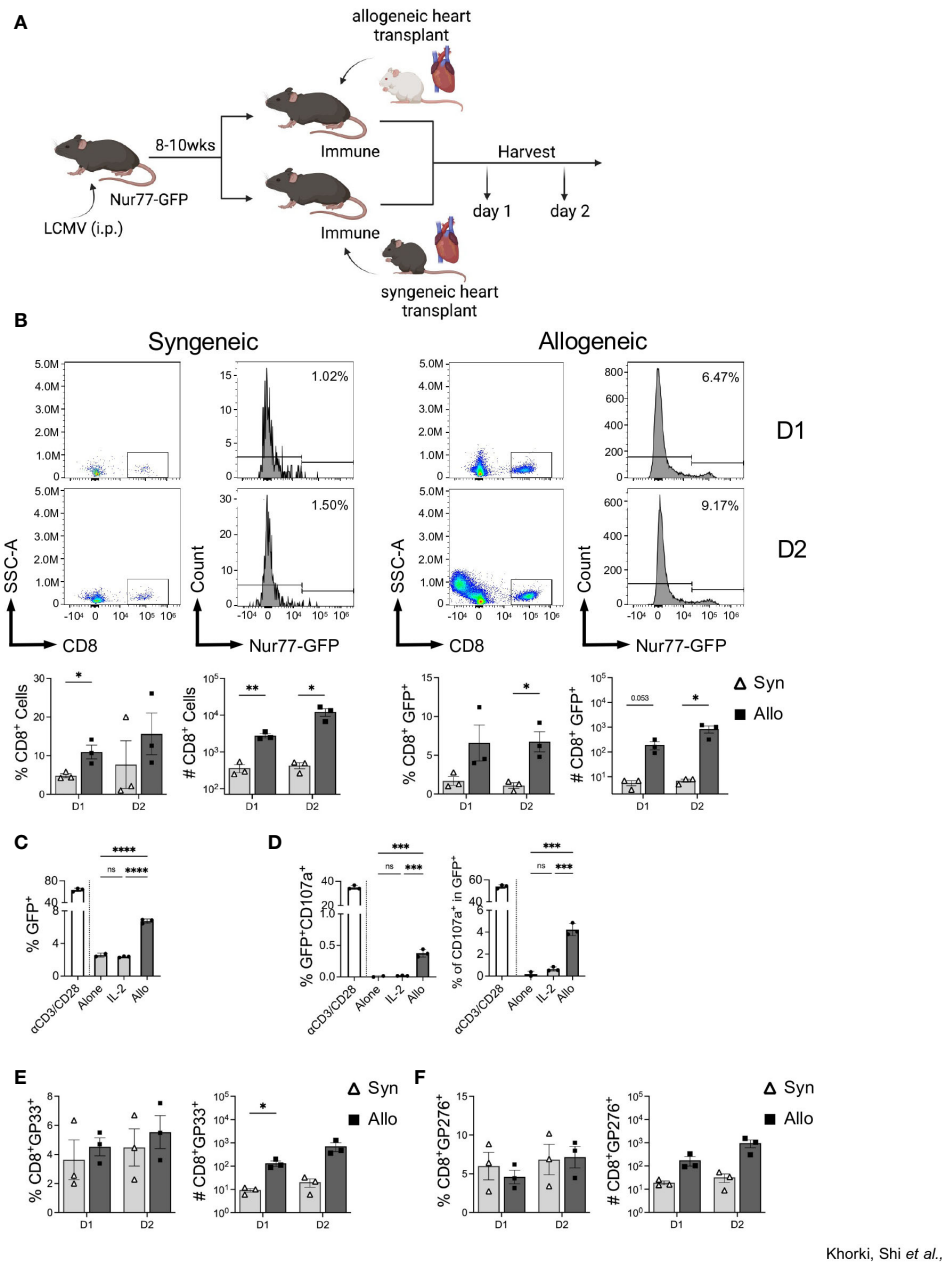
Infiltrating CD8^+^ T cells in LCMV immune mice respond to allogeneic, but not syngeneic, hearts. **(A)** Experimental outline showing LCMV-immune mice receiving either a syngeneic or allogeneic heterotopic heart transplantation, followed by sample collection at days 1 and 2 after transplantation Images were produced in BioRender ^(TM)^. **(B)** Representative flow plots of CD8^+^ T cells and Nur77-GFP expression within CD8^+^ T cells in heart allografts. **(C)** *In vitro* assay of GFP expression in sorted CD44^hi^ memory CD8^+^ T cells when cultured overnight with ɑCD3/CD28, IL-2, or allogeneic Balb/c T cell-depleted splenocytes. **(D)** Percentage of degranulating activated cells (GFP^+^CD107a^+^) (left) and percent of degranulating cells (CD107a^+^) in the GFP^+^ populations (right) of sorted memory CD8^+^ T cells after overnight *in vitro* culture. **(E)** D^b^GP33 tetramer and **(F)** D^b^GP276 tetramer expression in graft-infiltrating CD8^+^ T cells. Bar graphs show mean ± SEM. Statistical analyses were performed with student t-test between indicated groups (* p<0.05, ** p< 0.005, ***p<0.0005, **** p<0.00005).

To assess whether the increase in graft-infiltrating CD8^+^ T cells in LCMV-immune mice is an alloresponse or a non-specific overall response due to inflammation associated with ischemia-reperfusion injury in organ transplantation, immune mice were subjected to either a syngeneic (C56BL/6) or an allogeneic (Balb/c) heterotopic heart transplantation, and samples were collected on days 1 and 2 post-transplantation (**Figure 3A**). We observed more CD8^+^ T cell infiltration in mice that received allogeneic transplants relative to those receiving syngeneic transplants even as early as days 1 and 2 after transplant (**Figure 3B**). Further, we found a significant increase in the frequency of CD8^+^ T cells expressing GFP in mice receiving allogeneic, relative to syngeneic, heart transplants (**Figure 3B**). Further, overnight culture with IL-2 failed to drive an increase in GFP expression in sorted memory CD8^+^ T cells, whereas culture with Balb/c T cell-depleted splenocytes induced a significant increase in GFP expression (**Figure 3C**). Furthermore, allostimulation *in vitro* also induced degranulation, via CD107a expression, in the GFP population of sorted memory CD8^+^ T cells (**Figure 3D**). Again, stimulation with IL-2 did not result in CD107a expression (**Figure 3D**). Together, these data demonstrate that increased expression of GFP within LCMV-specific CD8^+^ T cells is likely due to TCR activation by alloantigens. Importantly, similar analyses performed in the lymph nodes of these mice reveal no significant differences in CD8^+^ T cell activation (**Supplemental Figure 3**), confirming the allograft-specific increase in CD8^+^ T cell GFP expression.

To verify that the recruited memory cells included those with LCMV specificity, we stained graft-infiltrating CD8^+^ T cells with two LCMV-specific tetramers, D^b^GP33 and D^b^GP276. No significant differences were seen between the proportion D^b^GP33^+^ and D^b^GP276^+^ CD8^+^ T cells (**Figures 3E, F**), despite a trend towards higher frequency and numbers of D^b^GP33^+^ CD8^+^ T cells seen at day 1 after allotransplantation (**Figure 3E**). Together, these data show that prior viral infection elicits viral-specific memory T cells that have the capacity to recognize alloantigens in the heart allograft and that their initial recruitment is preferential for cells with individual LCMV specificities.

### Viral-specific CD8^+^ T cells cross-reacting to heart allografts display epitope-specific variability

**Figure 4:**
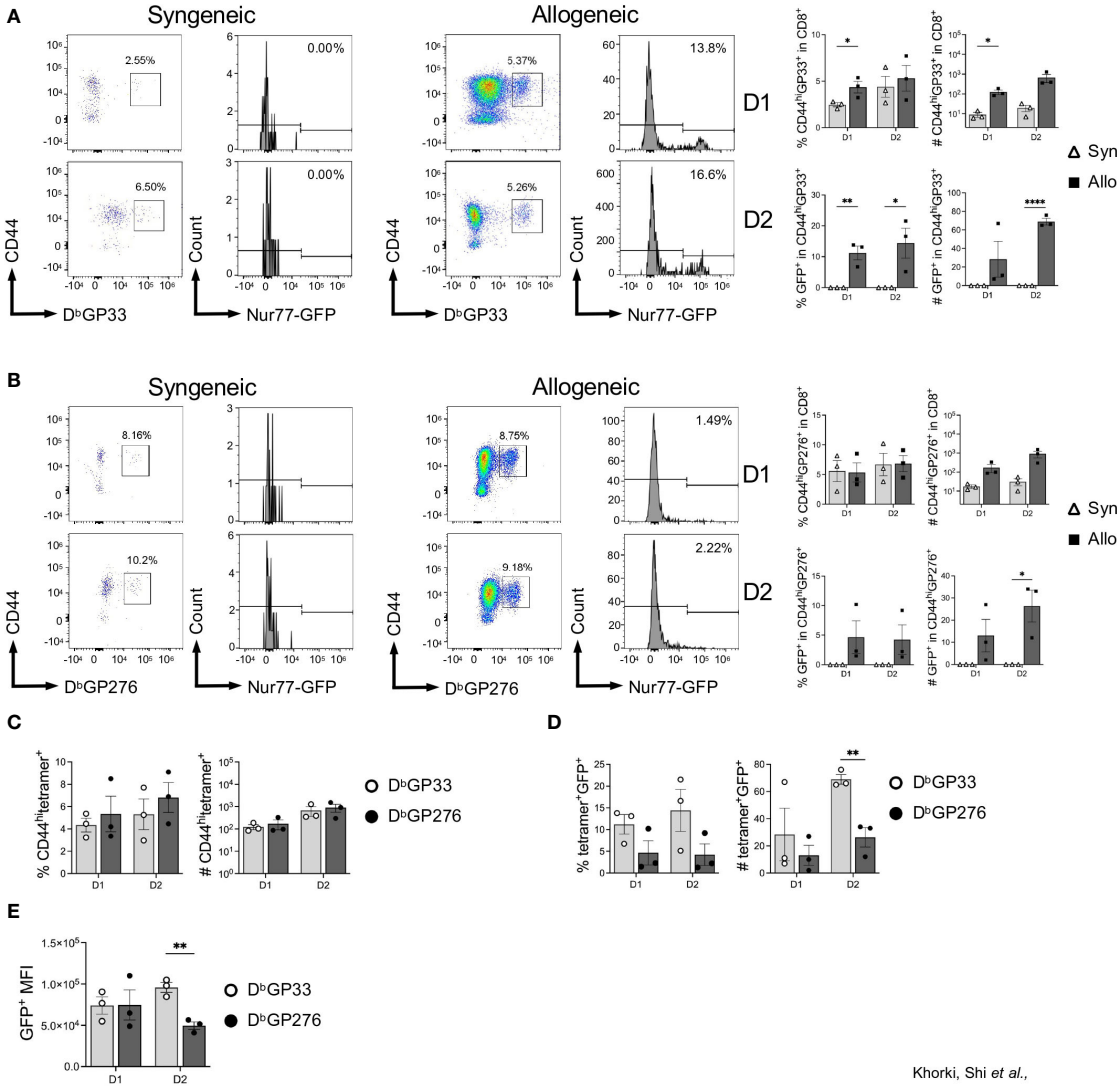
Viral-specific CD8+ T cells display epitope-specific variability in their ability to cross-react to heart allografts. **(A)** D^b^GP33-reactive and **(B)** D^b^GP276-reactive CD8+ T cell activation in syngeneic compared to allogeneic heart grafts. **(C)** Comparison of D^b^GP33- and D^b^276-tetramer positive cells in the CD8^+^CD44^hi^ population from allogeneic hearts. **(D)** Activation of D^b^GP33- and D^b^276-tetramer positive cells via Nur77-GFP expression in allogeneic hearts. **(E)** Mean fluorescence intensity (MFI) of GFP^+^ cells from D^b^GP33- and D^b^GP276-tetramer positive cells in allogeneic hearts. Bar graphs show mean ± SEM. Statistical analyses were performed with student t-test between indicated groups (* p<0.05, ** p< 0.005, **** p<0.00005).

Next, we determined whether graft-infiltrating LCMV-specific memory CD8^+^ T cells were capable of recognizing alloantigens on transplanted hearts by assessing their expression of Nur77-GFP. As expected, nearly all the LCMV-tetramer positive cells were CD44^hi^, indicating their memory status, and already by day 1 post-transplantation, there was a significant increase in CD44^hi^GP33^+^ cells seen in allogeneic hearts (**Figure 4A**). The fidelity of the Nur77-reporter was confirmed as a substantial fraction in allogeneic hearts were GFP^+^, while virtually none expressed GFP in syngeneic hearts (**Figure 4A**). Thus, in addition to their preferential recruitment to allogeneic heart grafts, a portion of these cells were also re-activated in the allograft, demonstrating their cross-reactivity to alloantigen *in vivo*.

Intriguingly, when we assessed recruitment and activation of D^b^GP276-specific CD8^+^ T cells, we observed far fewer GFP^+^ cells in allogeneic hearts (**Figure 4B**). Although there were no significant differences seen in the percentage of D^b^GP33- and D^b^GP276-positive cells in the CD8^+^CD44^hi^ populations (**Figure 4C**), D^b^GP33^+^ cells were proportionally more activated based on GFP expression compared to D^b^GP276^+^ cells, with significantly more of them infiltrating into heart allografts 2 days after transplantation (**Figure 4D**). Furthermore, when we assessed the mean fluorescence intensity (MFI) of the GFP^+^ populations of D^b^GP33-and D^b^GP276-positive cells, we observed that there was a significantly higher MFI in the D^b^GP33^+^ cells at day 2 after transplantation (**Figure 4E**), indicating increased TCR signaling strength in the D^b^GP33 cross-reactive cells. Combined, our data show that prior viral infection elicits significant increases in allograft infiltration by viral-specific T cells, and these viral-specific T cells display epitope-specific variability with their ability to cross-react with alloantigens.

### TCR sequencing reveals features associated with cross-reactivity

To ascertain the level of similarity among the TCRs from the different clonotype populations and identify any features in the CDR3 sequences distinguishing the potentially alloreactive clonotype populations from those that are not alloreactive, we sorted, sequenced, and analyzed the properties of CDR3 sequences from the following clonotype populations: CD44^low^GFP^-^, CD44^low^GFP^+^, GP33^+^GFP^-^, GP33^+^GFP^+^, GP276^+^GFP^-^, and GP276^+^GFP^+^.

We first examined the length and compositions of CDR3 sequences, including fractions of acidic/basic residues, hydrophobic residues, and aromatic residues (**Supplemental Figure 4**). We found that, for both the CDR3α and CDR3β sequences, although the degree of variability in each population differed, the median length was similar across all populations, differing by not more than 1 amino acid (excluding GP276^+^GFP^+^, for which only one paired sequence was obtained). Similarly, no clear differences were observed in amino acid composition between the GFP^-^ and GFP^+^ populations. We did notice that the median value for the percentage of acidic residues in the CDR3β sequences of the GP33^+^ populations (both GFP^+^ and GFP^-^) was greater than other populations, possibly reflecting differing antigen specificity rather than alloreactivity. A similar trend was observed for the percentage of aromatic residues.

**Figure 5:**
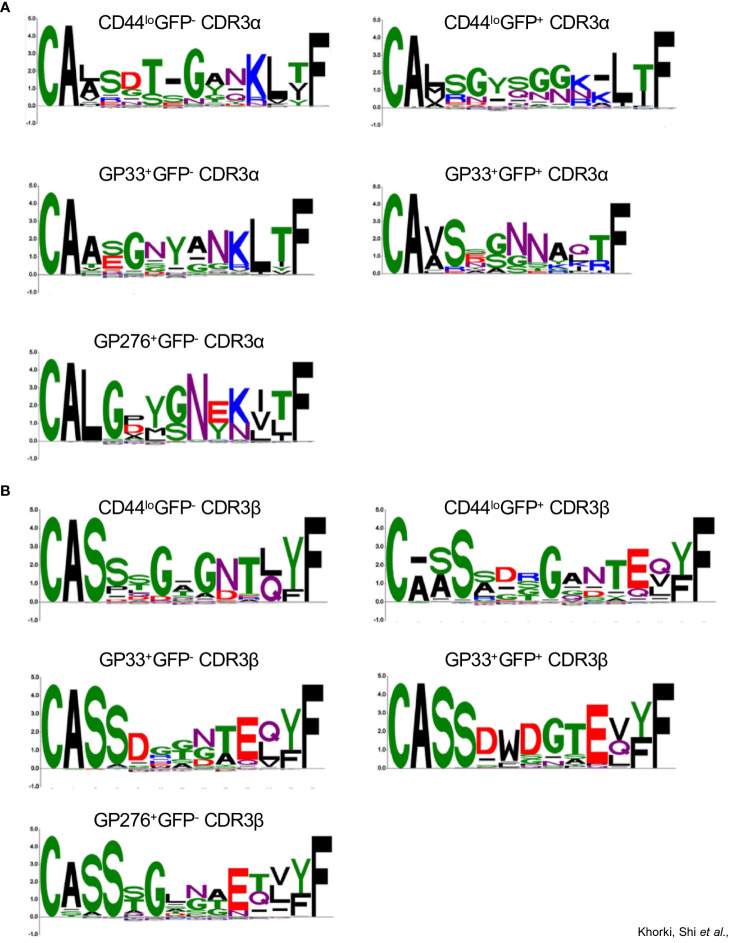
Sequence logo representations of **A)** CDR3α and **B)** CDR3β sequences of TCRs from the following clonotype populations: CD44^lo^GFP^-^(43 sequences), CD44^lo^GFP^+^ (70 sequences), GP33^+^GFP^-^ (66 sequences), GP33^+^GFP^+^ (21 sequences), and GP276^+^GFP^-^ (152 sequences).

For a more granular analysis, we constructed sequence logos using the CDR3 sequences of TCRs from the different clonotype populations (excluding GP276^+^GFP^+^ population as it contained only one paired CDR3 sequence). Here, notable differences in the CDR3 sequences were observed. For example, we observed that the CDR3α sequences of TCRs from the CD44^lo^GFP^+^ clonotypes, unlike those from the CD44^lo^GFP^-^ clonotypes, were enriched in a distinctive GYSGG motif in their core (**Figure 5A**). In contrast, the CDR3β sequences of most of the TCRs from both CD44^lo^ populations share a glycine in the middle of their sequences (**Figure 5B**). We also noticed differences between the non-viral reactive and viral reactive populations. Although the CDR3α sequences of most of the TCRs from viral reactive (GP33^+^ and GP276^+^) clonotypes and most of the TCRs from the CD44^lo^ clonotypes share the CA(A/L) and KLTF motifs at the beginning and end of their sequences, they differ in the amino acids found in the middle of the sequences, with G, N, Y and G, Y, G, N being prominent in GP33^+^ and GP276^+^ populations, respectively (**Figure 5A**). The conserved CA(V/A)S motif in the CDR3α sequences of the TCRs from the GP33^+^GFP^-^ and GP33^+^GFP^+^ clonotype populations suggests shared Vα-gene usage. However, less conservation is observed at the region of the sequences formed by the Jα segment. There is also an enrichment of a G/SNN motif in the CDR3α sequences from the GP33^+^GFP^+^ population (**Figure 5A**). Both populations also differ in their CDR3β sequences, with most TCRs from GP33^+^GFP^+^ population having D, W, D, and G in the middle of their CDR3 sequences (**Figure 5B**). Thus, it appears that cross-reactive CD8^+^ T cells are enriched in populations of cells whose TCRs have distinctive sequences that may endow their cross-reactivity.

## Discussion

Transplant recipients with high levels of pre-existing memory CD8^+^ T cells are more susceptible to acute cellular rejection (ACR) (15–17). To gain greater understanding of the cellular and molecular processes associated with this phenomenon, we developed a mouse model to interrogate the impact of pre-existing memory T cells on allograft rejection. This model revealed a number of important findings: (i) prior viral infection resulted in massively increased recruitment of CD44^hi^ CD8^+^ T cells into allogeneic, but not syngeneic, heart grafts; (ii) such enhanced recruitment was associated with substantially increased timing and severity of histologically-defined rejection; (iii) a substantial number of viral-specific memory CD8^+^ T cells cross-reacted to allogeneic, but not syngeneic, heart grafts; (iv) the cross-reactivity of viral-specific CD8^+^ T cells with alloantigen varied depending upon the viral epitope.

Mechanism(s) underlying increased recruitment of memory T cells to transplanted tissue is likely complex. CXCR3, highly expressed on quiescent anti-viral memory CD8^+^ T cells (30), likely contributes to their increased recruitment, although this remains to be definitively tested in our model. Importantly, CXCL9 and CXCL10, two ligands for CXCR3, are increased substantially within 24 hours after heart transplant (31, 32) and contribute to CD8^+^ T cell recruitment (31, 33–35). Several mechanisms likely contribute to increased expression of CXCL9/10 after transplantation. A major mechanism is likely ischemia reperfusion (I/R) injury, inherent in the transplantation of solid organs, which drives very early expression of the aforementioned chemokines (36–38). Although we found histologic evidence (small foci of epicardial damage) of I/R on days 1 and 2 post-transplant in LCMV-naïve mice, we did not see lymphocytic infiltration at this time, likely due to their lower expression of CXCR3 relative to memory T cells in immune mice. Indeed, compared to naïve mice transplanted with allogeneic hearts, immune mice transplanted with syngeneic hearts had substantially greater numbers of graft-infiltrating CD8^+^ T cells on day 1 after transplant. Thus, while I/R injury likely accounts for some of the initial infiltration, other mechanisms likely contribute. For example, recognition of allogeneic tissues (i.e., polymorphisms in CD47) by innate immune cells such as macrophages can promote early inflammation and organ rejection (39). Also, NK cells can also distinguish between self and non-self tissues (e.g., Ly49 molecules) and can contribute to increased expression of CXCL9 (31). Future work will determine the roles of NK cell and macrophage recognition to the enhanced T cell recruitment and activation observed in immune mice.

Beyond the preferential recruitment of memory T cells into allografts, this model allowed us to directly visualize viral-specific T cells that possess cross-reactivity to alloantigens based on expression of GFP emanating from the Nur77-GFP transgene. Although this phenomenon of viral infection increasing T cell alloreactivity has been known for decades (40–42), and may be specific only to some viruses (20, 23, 24), our approach allows us to examine this cross-reactivity in real-time *in vivo*, instead of relying on restimulation experiments *in vitro*. It is unlikely that GFP expression within viral specific T cells represents “bystander” activation, as we did not observe GFP expression after *in vitro* culture with IL-2, nor in syngeneic hearts, despite the recruitment of viral-specific T cells into those tissues. Further, another group elegantly showed that, even when present in the same tissue during infection, GFP expression was only observed in TCR transgenic T cells with specificity to antigen, not in TCR transgenic T cells with irrelevant specificity (43). While we do not exclude the possibility that viral-specific memory cells can be activated in a bystander fashion (i.e., without direct TCR stimulation), our data demonstrate that cross-reactivity can and does occur *in vivo*. Further, in naïve mice, we detected CD8^+^ T cells receiving TCR stimulation (assessed by GFP and CD69 expression) with similar kinetics observed in prior reports of T cell infiltration and rejection (32, 44). Further, we show that these cells are capable of degranulation, demonstrating their functionality. Thus, this model allows for the study of viral-specific CD8^+^ T cells with cross-reactivity to alloantigens as well as tracking and monitoring the *de novo* allo-response in naïve mice.

An unexplained conundrum is how TCRs with such fine specificity for peptides in the context of self-MHC molecules can also cross-react with alloantigens. One explanation posits that there is something unique about TCRs that possess cross-reactivity. For example, some evidence suggests that they may have shorter CDR3 regions that would prevent extensive contact with peptide/MHC and therefore possess less specificity (45). Alternatively, there are examples of molecular mimicry where the peptide presented in the context of allo-MHC is present in a similar fashion to the foreign peptide in the context of self-MHC (6, 46, 47). Our observations of sequence differences between the various sets of TCRs suggests a structural/biochemical basis for allospecific cross-recognition that studies of individual receptors and the ligands they recognize should illuminate.

Interestingly, we also found that the frequency of D^b^GP33-specific T cells that cross-reacted to allogeneic hearts was substantially higher than that observed for D^b^GP276-specific T cells. We think that the number of naïve precursors specific for the GP33-41 epitope have been predicted to be 3-4 times greater than for the GP276-286 epitope (300-400 vs ~100, respectively) (48, 49). Thus, the lower number of D^b^GP276-specific T cell precursors could translate into lower numbers of cross-reactive T cells. However, it could also be that the presentation of allo-peptides by K^d^, L^d^, or D^d^ that structurally mimic the presentation of the GP276-286 epitope in D^b^ to GP276-specific CD8^+^ T cells are far fewer than those that structurally mimic the presentation of the GP33-41 epitope in D^b^ to GP33-specific T cells (50, 51). Further work identifying peptides and allo-MHC responsible for stimulating these cross-reactive TCRs will help distinguish between these possibilities. Unfortunately, we obtained insufficient TCR sequences to determine if the structural changes observed in cross-reactive T cells specific for D^b^GP33 were conserved in T cells with specificity for D^b^GP276.

Our study also has implications for the impact of prior viral infections on transplant rejection. While a recent study from one of our co-authors showed that COVID-19 infection does not generally induce the production of donor-specific antibody (52), several studies have suggested an increased incidence of kidney rejection and graft-loss with severe COVID-19 infection, likely T cell mediated (53). It would be tempting to invoke cross-reactive T cells as an underlying mechanism, but several other non-mutually exclusive factors likely play significant roles. For example, to facilitate immunity to infection, a patient’s immunosuppression is either tapered or removed, which favors T cell-mediated rejection. In addition, viral infections can promote bystander activation of alloreactive T cells (23), especially if allografts themselves are infected and generate abundant local pro-inflammatory cytokine production. Another consideration is the potential T cell cross-reactivity to latent viruses such as EBV and/or CMV. As discussed below, EBV is known to generate cross-reactive CD8+ T cells in patients with certain HLA combinations, although their consequence to allograft rejection remains unclear. Compared to a quiescent memory population as seen in acute LCMV infection or an exhausted T cell population as seen with chronic LCMV infection, several studies, including from our group showed that MCMV-specific T cells are thought to continuously suppress MCMV reactivation in different tissues using different mechanisms (54–56). As other work has suggested a lack of cross-reactivity during mouse polyoma virus infection (23), it would be interesting to compare the impact of other chronic viral infections on T cell cross-reactivity, allograft rejection, and control of viral reactivation to determine the broader applicability of this concept.

Given that thymic selection must produce billions of T cells bearing unique TCRs with unknown utility, others have suggested that alloreactivity is simply a by-product of thymic selection (2, 3). This argument posits that alloreactive T cells never encounter peptides presented in the context of allo-MHC and are therefore never negatively selected against. Evidence for this argument comes from elegant experiments several decades ago that identified a public TCR specific for an EBV peptide bound to HLA-B8, which was present in all EBV seropositive HLA-B8^+^ individuals studied and displayed significant cross-specificity against HLA-B44.02 (7, 57). Strikingly, these authors uncovered an EBV seropositive patient that expressed both HLA-B8 and HLA-B44.02 alleles and this public TCR was lost (58). Together, these data suggest that cross-reactivity is the result of a lack of negative selection against peptides presented in the context allo-MHC, although this remains to be more definitively tested.

Beyond understanding basic immunologic tenets, there is significant clinical interest in understanding TCR cross-reactivity. While the development of immune suppressive drugs such as tacrolimus have greatly lessened rejection, they have also resulted in increased emergence of viral infections, which significantly increases the risk of allograft loss. With the recent success of viral-specific T cell (VST) therapies in bone marrow transplantation (BMT) (59), ongoing clinical trials using VSTs to treat infections in kidney transplant patients are emerging. However, additional consideration should be given when moving VST therapy from BMT patients into kidney transplant recipients. In BMT, VSTs are matched to recipient HLA, facilitating viral-peptide specific, self-HLA restricted responses, which are highly efficient at eliminating virally infected cells. However, in kidney transplant patients, VSTs are also matched to recipient HLA. While this is probably fine for CMV and EBV infection, as they primarily infect cells of recipient origin, it has the potential to be problematic for BK infection, as BK virus largely infects kidney cells, which are of donor origin. Further, given the success of tacrolimus in controlling ACR, HLA matching of kidney recipient and donor is no longer required (although the presence of donor specific antibodies will often prevent the transplantation of kidneys bearing certain HLA molecules). Many kidneys that are currently being transplanted are therefore “immunologically unprotected” by the immune system of their recipient as they have no HLA alleles in common. Thus, should these kidneys become infected with BK virus, viral clearance will rely on alloreactivity (or cross-reactivity), and in the case of VSTs, matching to the recipient will rely on either bystander activation or on cross-reactivity, which could range from being inefficient at best or trigger alloreactivity at worst. Further studies need to be performed on investigating the efficacy of matching VSTs to donor versus recipient (at least one allele) when treating patients with completely mis-matched kidneys for BK viral infection. However, we also realize that this is a complex issue as donor-matched VSTs could also have cross-reactivity to allo-HLA. Moreover, it is possible that donor matched VSTs could be a source of donor HLA that could stimulate an alloresponse. One solution might be to develop assays to screen potential alloreactivity within the VST pool as our data would suggest that this may vary widely depending upon donor HLA.

## Methods

### Procedures

7-8 week old Nur77-GFP transgenic mice (C57BL/6 background) were infected with 2x10^5^ pfu of LCMV via intraperitoneal injection. Uninfected Nur77-GFP mice were used as control. Approximately 8 weeks later, mice received either a syngeneic (C57BL/6 wild-type) or allogeneic (Balb/c wild-type) heart via heterotopic cervical heart transplantation (60). Mice were monitored daily, and heartbeat of the graft was assessed via manual palpation and visualization to determine function and viability of the graft. Animals were housed under specific pathogen-free conditions under the care of the Veterinary Services Facility at Cincinnati Children’s Hospital Medical Center. All experimental procedures were reviewed and approved by the Institutional Animal Care and Use Committee (IACUC) at the Cincinnati Children’s Hospital Research Foundation.

### Heart sample collection

On days 1 through 4 after transplant, mice were sacrificed. Mice were first perfused with 10mL basic salt solution (BSS) through their native heart, followed by an additional 10mL BSS perfusion of the transplanted heart. The transplanted heart was then excised, and both left and right ventricles were cut open to assess for major clots. Identified clots were then removed from the chambers as necessary, and the sample was then placed in BSS on ice. A single cell suspension was obtained from a modified cold-active protease tissue digestion protocol as previously described (61, 62). Debris from the sample was removed (Miltenyi Biotec), followed by red blood cell lysis (eBioscience). Cells were then counted and plated to prepare for staining.

### Lymph node sample collection

Following excision of the transplanted heart, lymph nodes (including brachial, axillary, submandibular, and/or mesenteric) were identified and collected in BSS. Samples were washed and strained through a 70uM strainer to obtain a single cell suspension. Cells were then counted and plated to prepare for staining.

### Cell staining

After blocking with 2.4G2 and Live/Dead Blue (Invitrogen) staining, cells were incubated with the GP33-PE tetramer (**Supplemental Table 1**) for 45 minutes at 4°C. Cells were then stained with a combination cocktail of the GP276-APC tetramer along with other surface markers (**Supplemental Table 1**) for 45 minutes at 4°C. Samples were washed and ran using spectral flow cytometry (Cytek Biosciences).

### Flow cytometry analysis

Flow cytometry analysis was performed using FlowJo (version 10.9.0). Cell populations of interest were analyzed accordingly (**Supplemental Figure 1**). All absolute counts analyzed are depicted as ‘per heart’.

### *In vitro* culture

Splenocytes from two LCMV-immune mice were sorted for memory CD8^+^ T cells using a B220^+^CD11c^+^CD11b^+^CD4^+^ dump gate and then selected based on high expression of CD44. Sorted cells were plated overnight either: alone, with αCD3/CD28 Dynabeads™ (Thermo Fisher Scientific), with 5ng/mL IL-2, or with T cell-depleted splenocytes from a Balb/c mouse. T cells were depleted using the MojoSort™ Mouse CD3 Selection Kit (BioLegend). 1uL/well of the CD107a antibody (**Supplemental Table 1**) was added to each well prior to overnight culture for assessment of degranulation. Following overnight culture, cells were stained and analyzed by flow cytometry as mentioned above.

### TCR sequencing and analysis

Three immune mice receiving allogeneic heart transplants were sacrificed at days 2 (n=2) and 3 (n=1) after transplantation. Hearts were digested as described above and pool together for cell staining. The FACSymphony S6 (BD Biosciences) was used to sort for the following 6 CD8^+^ T cell populations: CD44^hi^GP33^+^GFP^+^, CD44^hi^GP33^+^GFP^-^, CD44^hi^GP276^+^GFP^+^, CD44^hi^GFP276^+^GFP^-^, CD44^lo^Tetramer^-^GFP^+^, CD44^lo^Tetramer^-^GFP^-^. Samples with less than 3000 cells were topped off with purified B cells. These 6 samples were then processed for single-cell sequencing using the Chromium Next GEM 5’v2 Single Cell V(D)J Reagent Kit protocol. Libraries of enriched TCR sequences were created using the Single Index Kit Set A (10X Genomics) and sequenced on the NovaSeq 6000 (Illumina). Using Cell Ranger (v.6.1.2), raw files were demultiplexed and reads were aligned to the mouse reference genome package (mm10, GENCODEvM23). Loupe V(D)J (10X Genomics) was used to visualize and obtain TCR sequencing data. Sequences are deposited as NCBI Biosamples, accession numbers SAMN37531276, SAMN37531277, SAMN37531278, SAMN37531279, SAMN37531280, and SAMN37531281.

For TCR sequence analysis, we used TCRdist, an open-source Python package used for TCR sequence analysis (63), to generate sequence logo plots of the CDR3 sequences of the TCRs from the following clonotypes: CD44^lo^GFP^-^ (43 sequences), CD44^lo^GFP^+^ (70 sequences), GP33^+^GFP^-^ (66 sequences), GP33^+^GFP^+^ (21 sequences), and GP276^+^GFP^-^ (152 sequences). Not enough TCRαβ paired sequences were generated from GP276^+^GFP^+^ cells for analysis. For length and composition, we used the R package, alakazam (64).

### Histology

Following perfusion and excision of the transplanted hearts identified for histological analysis, hearts were bisected axially to visualize the right and left ventricles and placed in 10% formalin for 24-48 hours at room temperature before moving them into 70% ethanol. Samples were then processed for paraffin embedding using the Cincinnati Children’s Pathology Research Core. Slides were cut, and hematoxylin and eosin (H&E) staining was performed to visualize pathology. Representative H&E images were taken at 10x objective magnification using a Nikon Eclipse 80i microscope with Nikon DS-Fi3 camera and NIS-elements F camera software (Nikon).

Blinded histologic scoring of H&E slides were performed using the 1990 (65) and 2004 (66) International Society of Heart and Lung Transplantation (ISHLT) guidelines. Samples were then further described using the individual characteristics making up the rejection scores (**Table 1**).

### Statistical analysis

All statistical analyses, including student t-tests, were performed using GraphPad Prism (version 10.0.0).

## Data availability statement

The data presented in the study are deposited in the NCBI as Biosamples, accession numbers SAMN37531276, SAMN37531277, SAMN37531278, SAMN37531279, SAMN37531280, and SAMN37531281.

## Ethics statement

The animal study was approved by Institutional Animal Care and Use Committee at the Cincinnati Children’s Hospital Research Foundation. The study was conducted in accordance with the local legislation and institutional requirements.

## Author contributions

MK: Conceptualization, Data curation, Formal Analysis, Investigation, Methodology, Writing – original draft, Writing – review & editing. TS: Conceptualization, Data curation, Formal Analysis, Funding acquisition, Investigation, Methodology, Supervision, Validation, Writing – original draft, Writing – review & editing. EC: Data curation, Writing – review & editing. AB: Conceptualization, Supervision, Writing – review & editing. PC: Data curation, Investigation, Writing – review & editing. JP: Data curation, Formal Analysis, Writing – review & editing. MM: Data curation, Writing – review & editing. EW: Writing – review & editing. JM: Writing – review & editing. AF: Resources, Writing – review & editing. JK: Writing – review & editing. BB: Funding acquisition, Writing – review & editing. DH: Conceptualization, Funding acquisition, Methodology, Supervision, Writing – original draft, Writing – review & editing.

## Supplementary material

Supplementary Figure 1 Gating strategy for **(A)** CD8^+^ T cells was performed using an LCMV-immune mouse on day 2 after allogeneic heart transplant and **(B)** GFP using the native heart from a naïve Nur77-GFP transgenic mouse.

Supplementary Figure 2 A large majority of allograft-infiltrating CD8^+^GPF^+^ cells are also CD69^hi^. **(A)** Graph shows percentage of CD8^+^GFP^+^ that are CD69^hi^. Bar graphs show mean ± SEM. Statistical analyses were performed with student t-test between naïve and immune groups at each timepoint.

Supplementary Figure 3 No significant differences seen between lymph nodes obtained from LCMV-immune mice receiving allogeneic or syngeneic hearts. **(A)** Representative flow plots of CD8+ T cells and Nur77-GFP expression in naïve C57BL/6 WT (top) and Nur77-GFP (bottom) lymph nodes. **(B)** Representative flow plots of CD8^+^ T cells and Nur77-GFP expression within CD8^+^ T cells in brachial, axillary, and submandibular lymph nodes. **(C)** Comparisons between the percent of CD8^+^ T cells and Nur77-GFP expression within CD8^+^ T cells in lymph nodes of mice receiving allogeneic or syngeneic hearts. Bar graphs show mean ± SEM. Statistical analyses were performed with student t-test between groups.

Supplementary Figure 4 Analysis of length and amino acid compositions of **A)** CDR3α and **B)** CDR3β sequences from the following populations: CD44^low^GFP^-^ (Non-allo_Non-viral; lavender), CD44^low^GFP^+^ (Allo_Non-viral; purple), GP33^+^GFP^-^ (Non-allo_GP33; beige), GP33^+^GFP^+^ (Allo_GP33; coral), GP276^+^GFP^-^ (Non-allo_GP276; gray), and GP276^+^GFP^+^ (Allo_Non-viral; orange).

Supplementary Table 1 List of markers used for flow cytometry.
