# Supplementary figures and images for: Prior viral infection primes cross-reactive CD8+ T cells that respond to mouse heart allografts
Source: Front Immunol. 2023 Dec 8;14:1287546. doi: 10.3389/fimmu.2023.1287546 (PMC10748599; doi:10.3389/fimmu.2023.1287546)

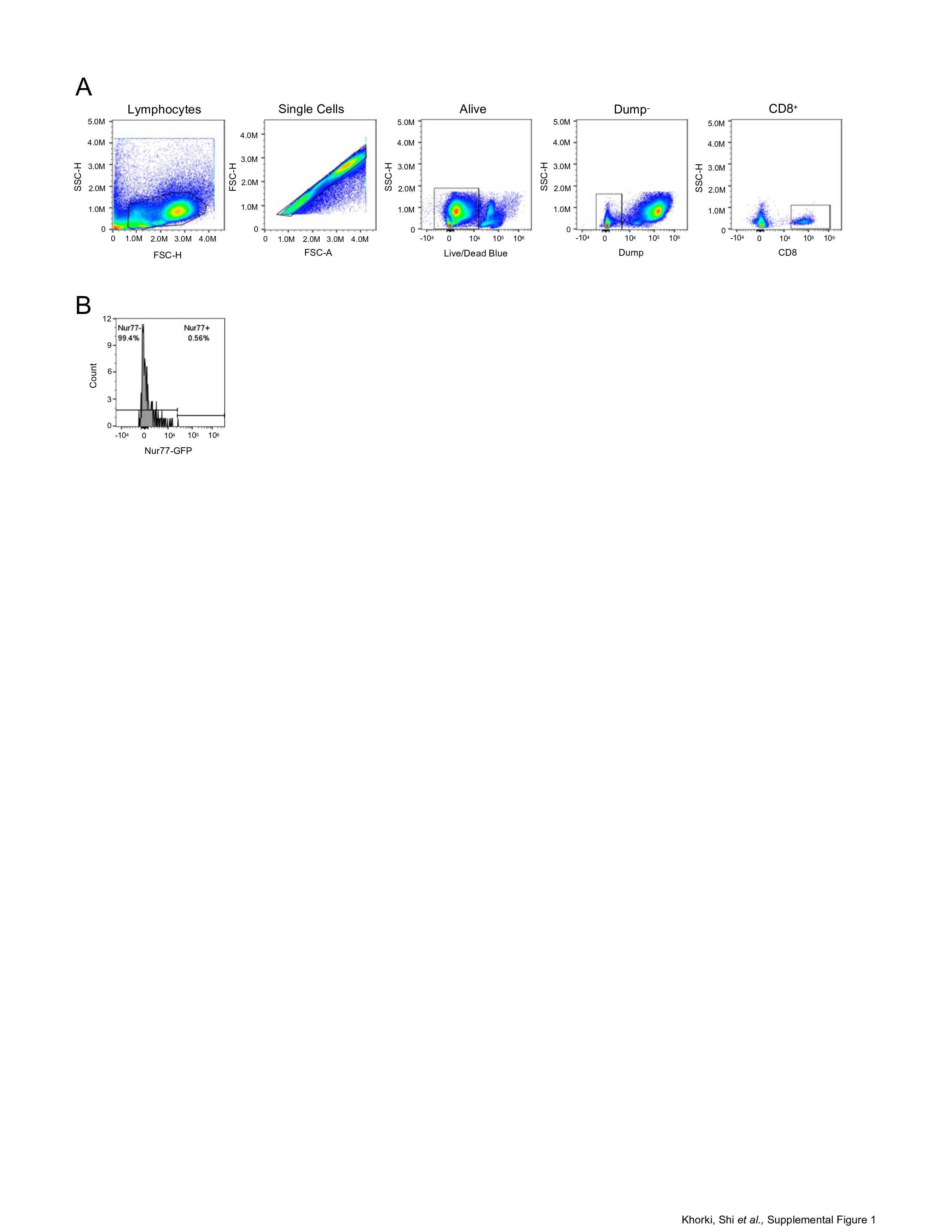

Supplement: Supplementary Figure 1 — Gating strategy for (A) CD8+ T cells was performed using an LCMV-immune mouse on day 2 after allogeneic heart transplant and (B) GFP using the native heart from a naïve Nur77-GFP transgenic mouse. [file Image_1.jpeg]

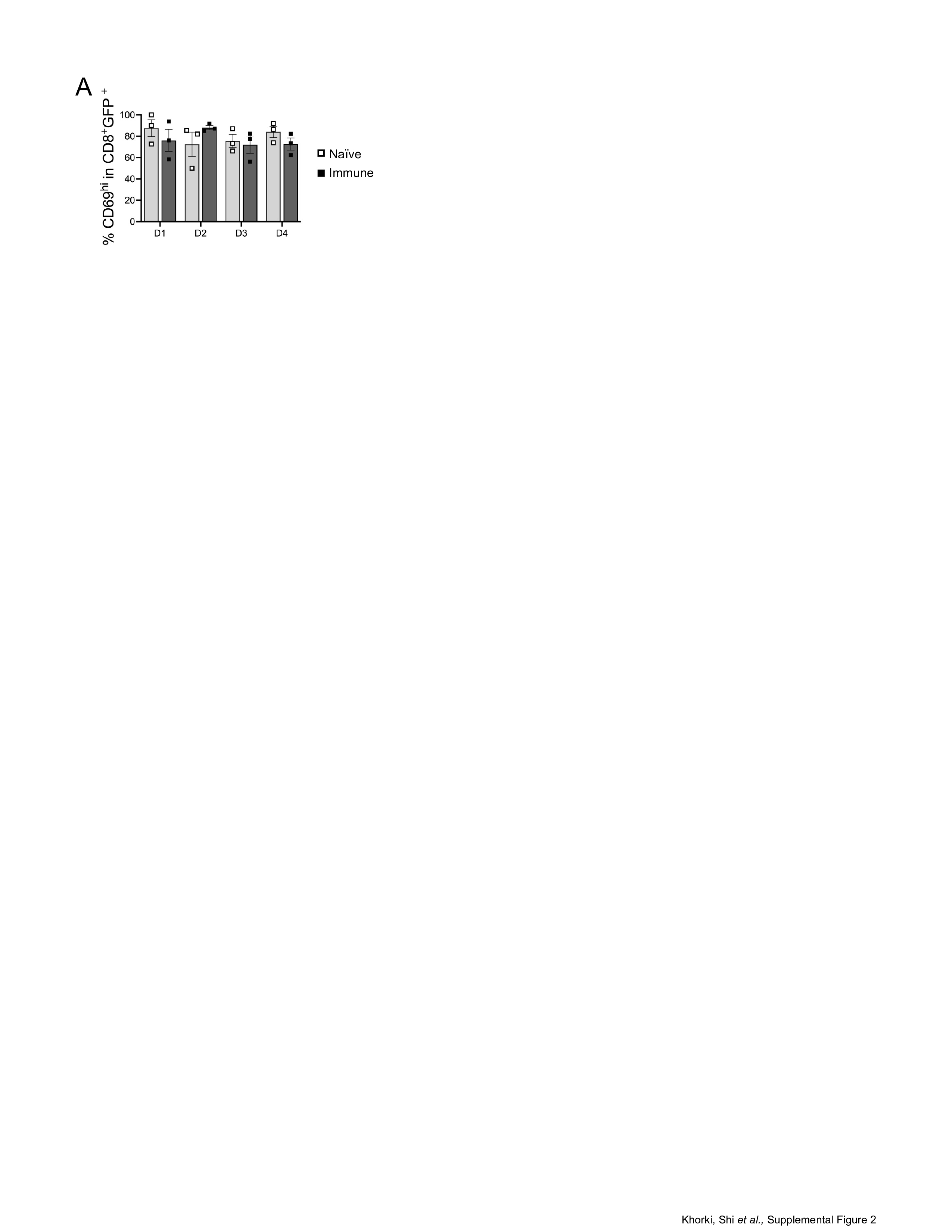

Supplement: Supplementary Figure 2 — A large majority of allograft-infiltrating CD8+GPF+ cells are also CD69hi. (A) Graph shows percentage of CD8+GFP+ that are CD69hi. Bar graphs show mean ± SEM. Statistical analyses were performed with student t-test between naïve and immune groups at each timepoint. [file Image_2.jpeg]

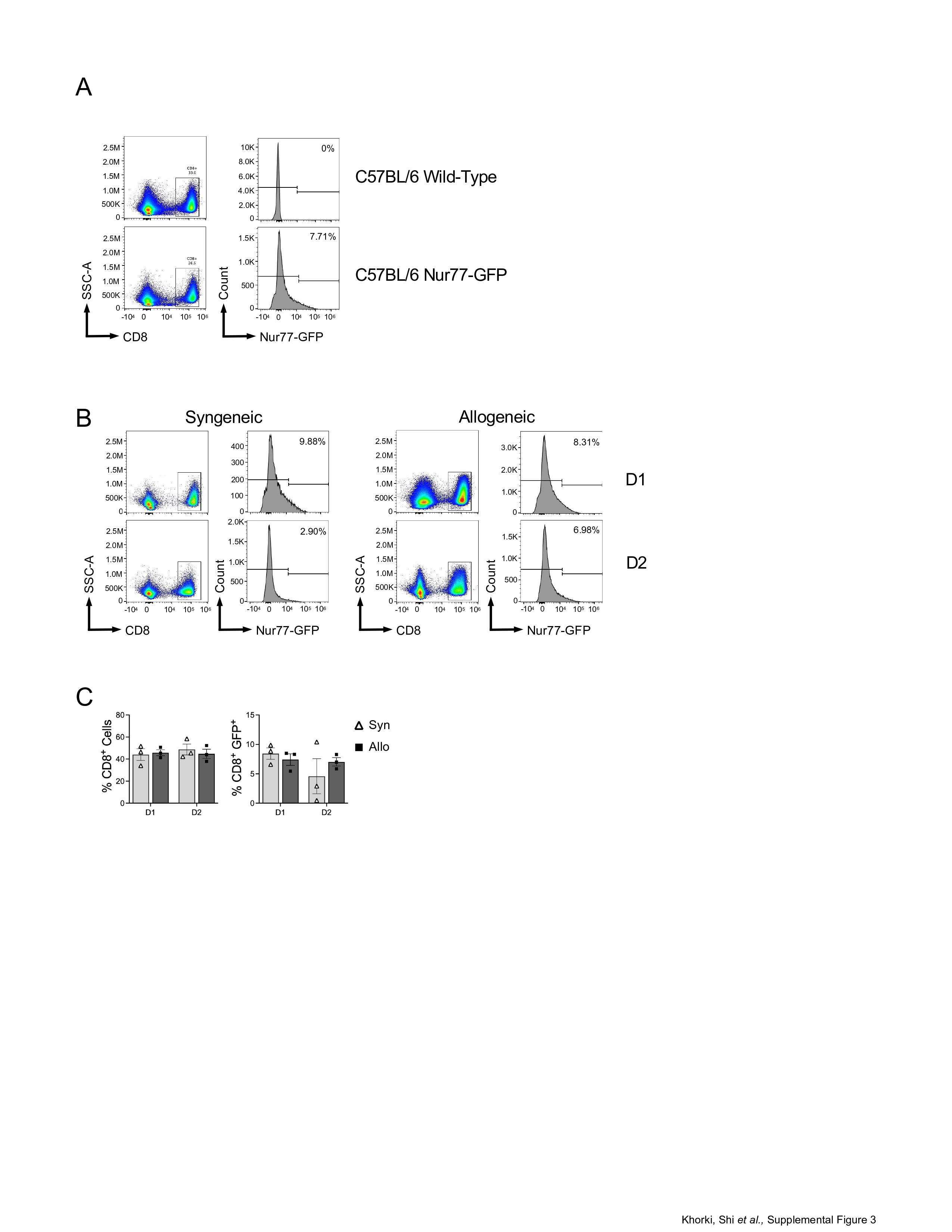

Supplement: Supplementary Figure 3 — No significant differences seen between lymph nodes obtained from LCMV-immune mice receiving allogeneic or syngeneic hearts. (A) Representative flow plots of CD8+ T cells and Nur77-GFP expression in naïve C57BL/6 WT (top) and Nur77-GFP (bottom) lymph nodes. (B) Representative flow plots of CD8+ T cells and Nur77-GFP expression within CD8+ T cells in brachial, axillary, and submandibular lymph nodes. (C) Comparisons between the percent of CD8+ T cells and Nur77-GFP expression within CD8+ T cells in lymph nodes of mice receiving allogeneic or syngeneic hearts. Bar graphs show mean ± SEM. Statistical analyses were performed with student t-test between groups. [file Image_3.jpeg]

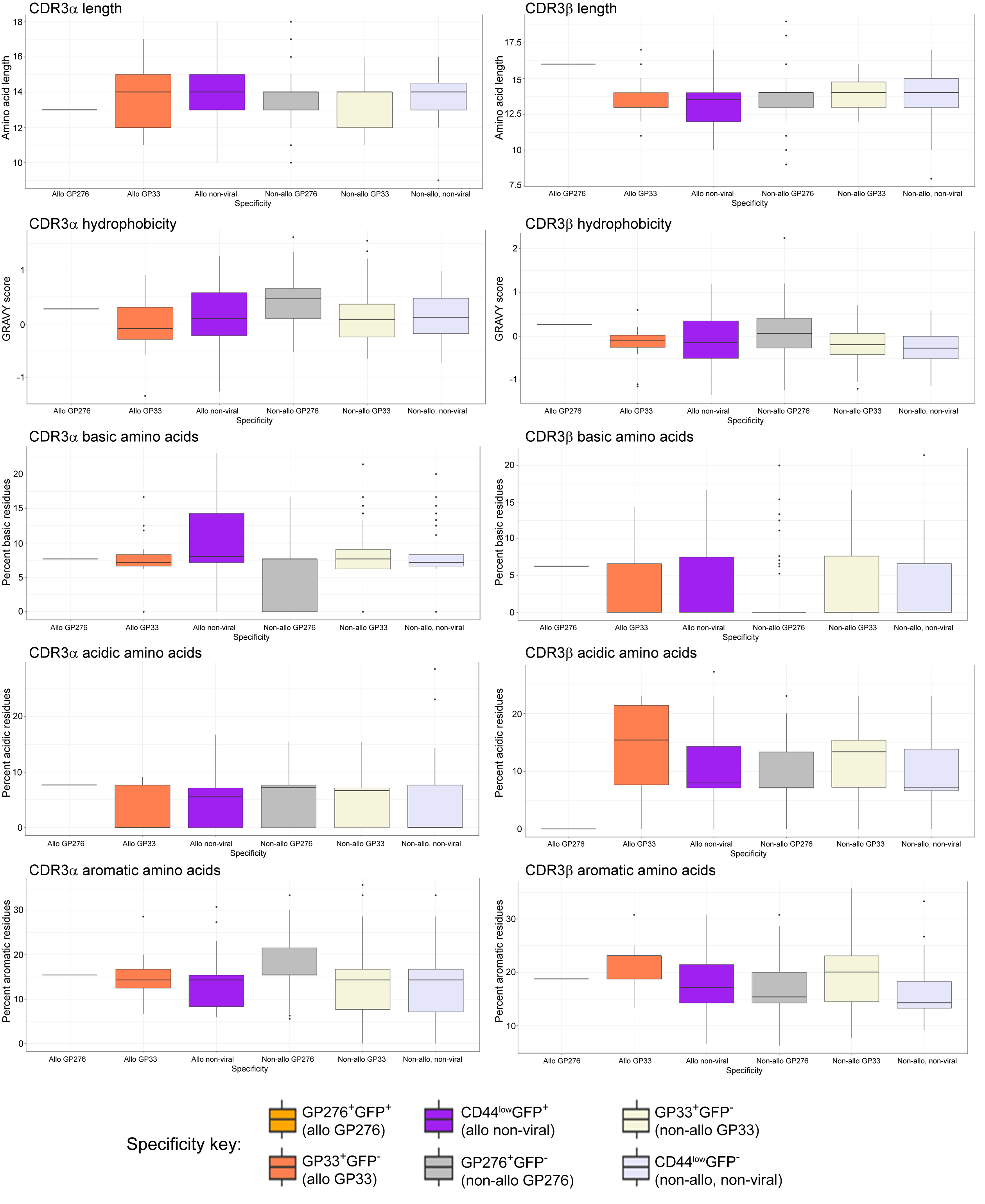

Supplement: Supplementary Figure 4 — Analysis of length and amino acid compositions of A) CDR3α and B) CDR3β sequences from the following populations: CD44lowGFP- (Non-allo_Non-viral; lavender), CD44lowGFP+ (Allo_Non-viral; purple), GP33+GFP- (Non-allo_GP33; beige), GP33+GFP+ (Allo_GP33; coral), GP276+GFP- (Non-allo_GP276; gray), and GP276+GFP+ (Allo_Non-viral; orange). [file Image_4.png]

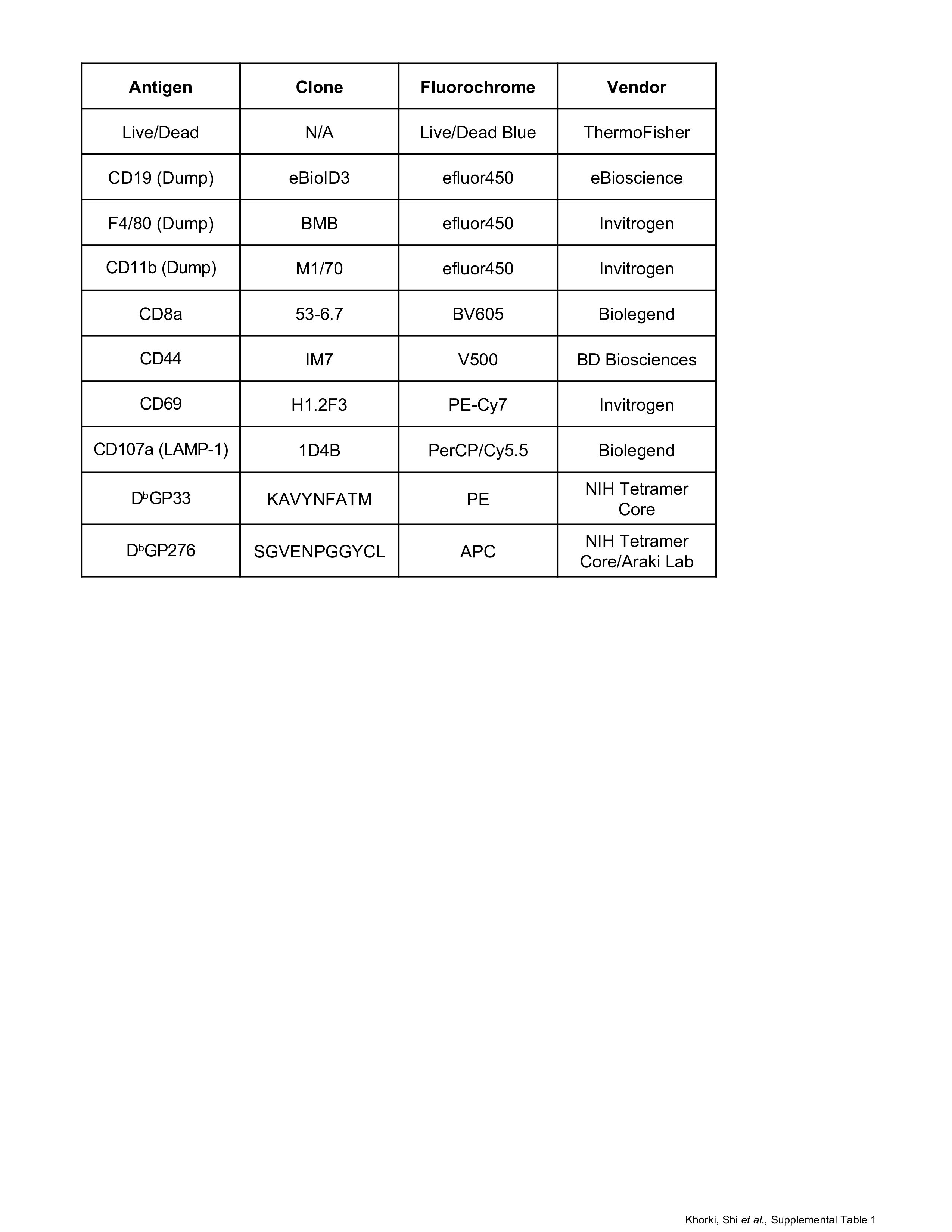

Supplement: Supplementary Table 1 — List of markers used for flow cytometry. [file Image_5.jpeg]
